# 维多珠单抗治疗糖皮质激素耐药急性肠道移植物抗宿主病2例报告并文献复习

**DOI:** 10.3760/cma.j.issn.0253-2727.2022.12.013

**Published:** 2022-12

**Authors:** 世春 高, 一梅 冯, 蕾 高, 佩艳 孔, 丽丹 朱, 嘉 刘, 路 王, 焕凤 刘, 曦 张

**Affiliations:** 陆军军医大学（第三军医大学）第二附属医院血液病医学中心，全军血液病中心，创伤、烧伤与复合伤国家重点实验室，重庆 400037 Medical Center of Hematology, Xinqiao Hospital of Army Medical University, State Key Laboratory of Trauma, Burns and Combined Injury, PLA Blood Disease Center, Chongqing 400037, China

异基因造血干细胞移植（allo-HSCT）是治疗多种良恶性血液病的重要方法，但存在移植物抗宿主病（GVHD）的风险，影响患者生存质量[1]–[2]。其中，急性GVHD（aGVHD）是allo-HSCT后的重要并发症，通常发生于移植后3个月内，主要受累器官为皮肤、肝脏、胃肠道[2]–[4]。其中60％的aGVHD患者有肠道症状，主要表现为厌食、腹泻、腹痛和出血等[5]。Ⅱ～Ⅳ度aGVHD的一线治疗为糖皮质激素，有效率近50％，有效患者中仅有1/3可持续缓解[6]–[7]，肠道受累患者对糖皮质激素反应更差、死亡率更高[8]。目前，糖皮质激素耐药肠道aGVHD的二线治疗药物众多，但缺乏稳定有效的治疗药物，严重肠道GVHD患者2年生存率不到20％[9]–[11]。近年研究发现，供者T淋巴细胞表面α4β7整合素是介导其向肠道淋巴组织迁移的重要信号分子[12]，参与肠道aGVHD的发生。维多珠单抗（Vedolizumab）是针对淋巴细胞表面α4β7整合素的人源化单抗，最初用于炎症性肠病（IBD）的治疗，后有研究发现其可以通过抑制初始和活化的T淋巴细胞向肠道相关淋巴组织的迁移来治疗肠道aGVHD[13]。我们应用维多珠单抗治疗2例糖皮质激素耐药肠道aGVHD患者并获得满意疗效，报告如下。

## 病例资料

例1，女，34岁，于2020年1月9日因鼻咽部新生物行病理活检及免疫组化、全身肿瘤PET/CT诊断为结外NK/T细胞淋巴瘤ⅣB期。先后于2020年1月18日、2月26日在我院肿瘤科行IMEP方案（异环磷酰胺+依托泊苷+甲氨蝶呤+泼尼松）化疗，2疗程后PET/CT评价为部分缓解（PR），继续行鼻咽部局部放疗1月余，此后未再行治疗。2021年3月11日出现疾病复发伴骨髓侵犯，转入我科予以P-GeMox方案（吉西他滨+奥沙利铂+培门冬酶）治疗，4月26日疾病进展伴不规则发热（体温最高39.4 °C），血常规：WBC 2.04×10^9^/L，中性粒细胞绝对值（ANC）1.47×10^9^/L，HGB 69 g/L，PLT 40×10^9^/L；凝血酶原时间（PT）17.8 s（参考值9.4～12.5 s），活化部分凝血活酶时间（APTT）51.9 s（参考值25.1～36.5 s），纤维蛋白原（FBG）0.53 g/L（参考值2.00～4.00 g/L）；肝功：丙氨酸转氨酶（ALT）94 U/L（参考值7～50 U/L），天冬氨酸转氨酶（AST）100 U/L（参考值13～40 U/L），碱性磷酸酶（ALP）634 U/L（参考值35～135 U/L），铁蛋白>2 000 µg/L（参考值5～365 µg/L）；腹部超声：脾大（约5.2 cm，上下径16.3 cm）。予以依托泊苷联合地塞米松治疗，效果欠佳，加用环磷酰胺联合长春地辛、培门冬酶、西达本胺治疗，6月2日腹部超声：肝大（左叶前后径6.3 cm，上下径12.0 cm，肝右叶斜径15.5 cm，肋缘下超过4.0 cm）、脾大（厚9.1 cm，上下径24.0 cm）；浅表淋巴结超声：双侧腋窝淋巴结肿大，皮质增厚（CDFI可见短棒状血流信号）。6月9日血常规：WBC 1.01×10^9^/L，ANC 0.64×10^9^/L，HGB 58 g/L，PLT 19×10^9^/L；PT 17.9 s，APTT 47.4 s，FBG 0.74 g/L；铁蛋白10 933 µg/L；6月9日、6月23日予以DEP方案化疗，后加用芦可替尼10 mg/d口服维持治疗。7月7日PET/CT提示疾病部分缓解。后患者噬血细胞综合征再次进展，于7月13日行同胞全相合造血干细胞移植，采用白消安（BU）+环磷酰胺（CTX）+依托泊苷预处理方案，预处理过程中出现高热、脾脏进行性增大（厚7.8 cm，上下径22.0 cm），加用甲泼尼龙（80 mg/d）、吉西他滨（2 g·m^−2^·d^−1^×3 d）、芦可替尼（10 mg/d）、兔抗人胸腺细胞免疫球蛋白（2.5 mg/kg）、重组抗CD25人源化单克隆抗体（1 mg/kg）；采用环孢素A+霉酚酸酯（MMF）预防GVHD。7月20日回输供者外周血干细胞（单个核细胞13.6×10^8^/kg，CD34^+^细胞9.2×10^6^/kg）。+17 d粒细胞植入，+35 d血小板植入、体温恢复正常，甲泼尼龙缓慢减量至40 mg/d。8月12日复查骨髓示：增生减低，未查见异常表型T淋巴细胞，植入XY 100％。8月13日停用霉酚酸酯，8月16日复查腹部超声示：肝脾肿大（脾厚5.6 cm，上下径18.0 cm）。8月20日停环孢素A。患者于8月20日开始腹泻（黄色水样便5次，共400 ml），大便常规+隐血未见异常，大便细菌培养及真菌培养未见异常。对症处理效果差，日腹泻量进行性增加至1 600 ml，考虑肠道aGVHD（Ⅲ度）。予以环孢素A 100 mg/d、重组抗CD25人源化单克隆抗体2剂（1 mg/kg，间隔3 d），患者腹泻、腹痛加重（每日排便20余次，量约2500 ml），予以禁食水、停用环孢素A改为他克莫司。9月1日患者开始排血便，予以艾司奥美拉唑及生长抑素抑酸止血治疗，维持他克莫司血浓度10 µg/L。9月2日使用第3剂重组抗CD25人源化单克隆抗体（1 mg/kg），但患者出现剑突下、脐周及右下腹疼痛、腹部膨隆、肝脾肿大，腹部超声示脾大（厚5.4 cm、上下径16.8 cm）。9月6日腹泻29次，腹泻量410 ml，细胞因子检测提示IL-6 180.40 pg/ml，予以托珠单抗320 mg治疗及间充质干细胞输注（1×10^6^/kg每周1次，共4次），患者每日腹泻15～21次，量500～1 000 ml。9月9日给予第1剂维多珠单抗300 mg治疗，甲泼尼龙减量至30 mg/d，9月13日患者不能耐受饥饿引起胃痛恢复流质饮食，9月23日使用第2剂维多珠单抗300 mg，腹泻次数减至每日6次，量500～1 000 ml。10月3日患者腹痛、腹泻再次加重（腹泻15次，量1 000～1 600 ml）。10月7日EB病毒核酸检测6.9×10^4^ IU/ml，10月8日予以利妥昔单抗500 mg治疗。10月9日、10月12日再次给予重组抗CD25人源化单克隆抗体，患者腹泻量下降至500 ml（黄色稀水便，大便隐血阳性），期间排出少许坏死肠道黏膜组织。10月18日结肠内镜示：末端回肠黏膜糜烂（小肠aGVHD？），慢性结肠炎。加用美沙拉嗪并给予第3剂维多珠单抗治疗，留置胃管、肠内及肠外营养。10月20日复查EB病毒核酸阴性、血巨细胞病毒（CMV）阴性。腹部超声：脾大（厚4.1 cm，上下径14.8 cm）。11月15日患者大便恢复正常。复查腹部超声：肝脏未见异常，脾大（厚3.4 cm，上下径12.6 cm）。患者顺利出院。2022年1月12日全身PET/CT示完全缓解。2月21日血常规：WBC 3.55×10^9^/L，ANC 2.92×10^9^/L，HGB 73 g/L，PLT 55×10^9^/L。患者后因贫血、肺部感染门诊随访。3月2日（移植后8个月）患者因肺部重症感染、感染性休克、多器官功能衰竭死亡。

例2，女，29岁，2021年1月因乏力、牙龈出血就诊，诊断为：急性髓系白血病M2a型伴CEBPA突变（37.6％）、NRAS突变（6.9％）、WT1突变（43.9％）、BCORL1突变（50.4％）、CSF3R突变（13.8％）以及FLT3-ITD突变（20％）。予以IA方案（去甲氧柔红霉素+阿糖胞苷）诱导化疗后达到形态学完全缓解，流式细胞术微小残留病（FCM-MRD）阳性。经IA方案巩固化疗2疗程后复查完全缓解，FCM-MRD阴性。2021年6月8日行非血缘供者造血干细胞移植（男供女，HLA 9/10相合，单个核细胞8.2×10^8^/kg，CD34^+^细胞6×10^6^/kg），采用BU+CTX+Ara-C+兔抗人胸腺细胞免疫球蛋白方案进行移植预处理，以环孢素A、霉酚酸酯联合短程小剂量甲氨蝶呤预防GVHD。造血重建前，患者并发急性上消化道出血，对症处理后好转。7月4日（+19 d）粒细胞及血小板植入。7月19日复查骨穿：AML-CR，FISH探针法检测植入XY占99％。此时，患者出现尿频、尿痛，腹泻，排黄色稀水便5次（量约360 ml），复查血CMV 268 IU/ml（参考值<400 IU/ml），BK病毒>5.00×10^8^（参考值<5.00×10^3^）。予以水化碱化、抗病毒、抗感染治疗后尿路刺激征好转，但大便量以及性状无好转，加用甲泼尼龙60 mg/d以及芦可替尼（10 mg/d）加强aGVHD治疗。7月21日出现胸背部及双上肢皮肤皮疹（约占全身体表面积40％），加用2剂重组抗CD25人源化单克隆抗体（1 mg/kg，间隔2 d）治疗，7月24日出现剧烈腹痛（腹泻量约160 ml），7月26日皮疹消失，但腹泻量增加至500 ml，考虑肠道aGVHD加重，加用布地奈德胶囊、第3剂重组抗CD25人源化单克隆抗体，并将环孢素A改为他克莫司，患者腹痛仍无减轻，日腹泻量增加至1 000 ml。8月6日加用第1剂维多珠单抗300 mg，腹泻量明显减少（90～270 ml），腹痛明显好转。2周后予以第2剂维多珠单抗300 mg，腹痛完全消失，大便性状恢复正常。2022年9月26日骨穿提示本病完全缓解，流式细胞术MRD阴性，FISH探针法检测植入XY占100％。2022年10月（移植后14个月）血常规：WBC 5.07×10^9^/L，ANC 3.14×10^9^/L，HGB 121 g/L，PLT 156×10^9^/L。患者维持无病、无GVHD生存。

## 讨论及文献复习

aGVHD的一线治疗是糖皮质激素[14]–[16]，然而35％～50％的患者对糖皮质激素耐药[17]–[18]。目前对于糖皮质激素耐药aGVHD的一线推荐药物为芦可替尼[15],[19]。aGVHD的二线治疗包括：甲氨蝶呤、霉酚酸酯、IL-2受体单抗、阿仑单抗、抗胸腺细胞球蛋白（ATG）、依那西普、间充质干细胞以及英夫利昔单抗[16]。其余的新治疗方法有粪菌移植、贝伦妥单抗及维多珠单抗等[20]。本研究2例患者使用糖皮质激素、巴利昔单抗、他克莫司、CD25单抗及芦可替尼治疗后效果差，加用维多珠单抗治疗后腹痛、腹泻好转，提示维多珠单抗可能在钙调磷酸酶抑制剂基础上明显减轻肠道aGVHD的症状。

维多珠单抗作为一种人源化的α4β7整合素单克隆抗体，主要通过特异性阻断定位于胃肠道和胆道系统内皮细胞中α4β7整合素与黏膜地址素细胞黏附分子-1（MAdCAM1）的结合，抑制供者活化T细胞向肠道内皮细胞归巢，而不作用于中枢神经系统[21]–[22]。在炎症性肠病中有效。黏膜地址素细胞黏附分子-1（MAdCAM1）属于免疫球蛋白超家族，在肠系膜淋巴结的高内皮小静脉和固有层的Peyer's斑块及毛细血管后小静脉上表达，Peyer's斑块是引起肠道aGVHD的抗宿主细胞毒T细胞活化的关键[23]。

MAdCAM1不仅是α4β7整合素的主要配体，同时也是L-选择素的配体[24]。α4β7整合素在初始B淋巴细胞及T淋巴细胞低表达，在分泌IgA免疫球蛋白的浆细胞、记忆T淋巴细胞及活化的肠上型CD4^+^T淋巴细胞高表达。同样也表达在NK细胞、活化的单核细胞、巨噬细胞、嗜酸粒细胞及树突状细胞[25]。因此，维多珠单抗目前获准用于治疗克罗恩病和溃疡性结肠炎。基于aGVHD发生机制，抑制T细胞诱导的炎症反应可能是一种很有前景的方法。2016年Fløisand等[26]应用维多珠单抗300 mg治疗6例糖皮质激素耐药的肠道aGVHD患者，所有患者在治疗7～10 d内腹痛和水样泻均好转。2019年Fløisand等[27]报告了维多珠单抗治疗糖皮质激素耐药胃肠aGVHD的回顾性研究结果，维多珠单抗首剂治疗后8周的总反应率为64％，其中28％的患者完全缓解，6个月总生存率54％，1年总生存率47％，最严重的不良反应为4级败血症，而且维多珠单抗组较其余免疫抑制剂组本病复发率更低。2021年Fløisand等[28]报告了一项不同剂量维多珠单抗治疗糖皮质激素耐药肠道aGVHD安全性及有效性的Ⅱa期研究结果，显示维多珠单抗在肠道GVHD疾病晚期有可能无法发挥足够快的作用，从而使受损的胃肠道上皮充分恢复，并防止与疾病有关的并发症。有研究显示，如果发生足够的组织损伤，T细胞迁移到胃肠道内皮细胞的重要性是有限的，而α4β7整合素已经不再是引起GVHD的主要因素[29]。由此可见，维多珠单抗可能不会在疾病晚期发挥显著的治疗作用。Fu等[30]最近的一项研究证实了这一假说，该研究强调了α4β7整合素与黏膜地址素细胞黏附分子-1相结合在早期向肠道募集供者T细胞中起关键作用。Danylesko等[31]关于维多珠单抗治疗肠道aGVHD的回顾性分析结果显示，维多珠单抗治疗7～10 d后总反应率79％，其中完全缓解率、部分缓解率分别为为28％、52％，6个月、1年的总生存率分别为41.4％、27.5％，越早使用有效率越高。以上研究都提示维多珠单抗治疗aGVHD采用早期预防、抢先及初始治疗策略可能使患者更多收益。

Chen等[32]于2019年发表了一项应用维多珠单抗预防GVHD的Ⅰb期临床研究结果，患者对标准预防GVHD方案基础上加入300 mg维多珠单抗能耐受且不影响植入，肠道aGVHD发生率明显下降。2021年Ibrahimova等[33]关于儿童和青年肠道aGVHD中α4β7整合素表达的研究，结果显示肠aGVHD出现症状前1周α4β7整合素表达上调；7例糖皮质激素耐药肠道aGVHD患者中有4例维多珠单抗有效，1例患者在移植后28天完全缓解，2例部分缓解，1例无治疗反应，未观察到维多珠单抗的不良反应作用。2021年Isshiki等[34]使用维多珠单抗治疗3例儿童肠道aGVHD患者，2例有效，均无严重不良反应。2022年Rosa等[35]应用维多珠单抗挽救治疗3例糖皮质激素耐药肠道aGVHD儿童患者，1例完全缓解，1例死于本病复发，1例死于GVHD。以上研究显示维多珠单抗对于儿童是安全有效的。

然而，另一些研究结果截然相反，Bukauskas等[36]使用维多珠单抗治疗5例糖皮质激素耐药的aGVHD患者，所有患者都死于GVHD合并感染，仅仅在2例患者中观察到部分缓解，治疗失败原因可能是肠道aGVHD与皮肤及肝脏aGVHD并存且维多珠单抗作为3、4线挽救治疗选择，提示维多珠单抗适用于单纯肠道aGVHD的早期治疗。Coltoff等[37]应用维多珠单抗300 mg治疗9例糖皮质激素耐药肠道aGVHD患者，仅1例生存超过60 d，7例死于感染。

本组2例患者的临床治疗经过显示维多珠单抗能够明显改善肠道aGVHD症状。维多珠单抗预防性及早期应用能否提高肠道aGVHD的治愈率以及对患者感染及长期生存的影响尚需继续研究。
